# Mesothelioma of the testis and nephrotic syndrome: a case report

**DOI:** 10.1186/1752-1947-3-7248

**Published:** 2009-06-05

**Authors:** Justine Bacchetta, Dominique Ranchère, Frédérique Dijoud, Jean-Pierre Droz

**Affiliations:** 1 Departments of Medical Oncology and Pathology, Centre Léon Bérard, Lyon and Université Claude Bernard Lyon 1, France; 2 Department of Pathology, Hôpital Femme Mère Enfant, Bron and Université Claude Bernard Lyon 1, France

## Abstract

**Introduction:**

Paraneoplastic glomerulopathies are rare manifestations of neoplastic disease to be distinguished from iatrogenic renal damage. Solid tumors are preferentially associated with membranous nephropathy, whereas Hodgkin's lymphomas are associated with minimal change disease.

**Case presentation:**

We report a 63-year-old Caucasian male diagnosed with a mesothelioma of the tunica vaginalis testis who, secondary to this, also presented with a nephrotic syndrome due to minimal change disease. In the present case, the paraneoplastic etiology of the nephrotic syndrome can be discussed on four unusual elements: minimal change lesions were found; the glomerulopathy was very sensitive to corticosteroids; the nephrotic syndrome occurred 11 months after the diagnosis of the primary malignancy, but concomitantly with the recurrence; and the nephrotic syndrome did not decrease with tumor control and did not recur when the mesothelioma escaped treatment. No other etiologies could nevertheless explain this phenomenon.

**Conclusion:**

Paraneoplastic nephrotic syndrome is often associated with membranous nephropathy in patients with solid tumors, especially in patients with lung and gastrointestinal tract neoplasia. The management of these patients is associated with a symptomatic treatment such as sodium and water restriction, diuretics and ACE inhibitors and a prophylaxis of specific complications of nephrotic syndrome including thromboembolism, infections and lipid abnormalities. Treatment of neoplasia must be undertaken rapidly, treatments must be regularly analyzed and drugs binding to albumin may be used with precaution.

## Introduction

The term 'paraneoplastic syndrome' refers to clinical manifestations not directly related to tumor burden, invasion or metastasis, but caused by the secretion of tumor cell products such as hormones, cytokines, growth factors and tumor antigens. Paraneoplastic glomerulopathies are rare manifestations of neoplastic disease to be distinguished from iatrogenic renal damage. Solid tumors are preferentially associated with membranous nephropathy, whereas Hodgkin's lymphomas are associated with minimal change disease. Paraneoplastic glomerulopathies are well known entities rarely associated with mesothelioma.

## Case presentation

**Figure 1:**
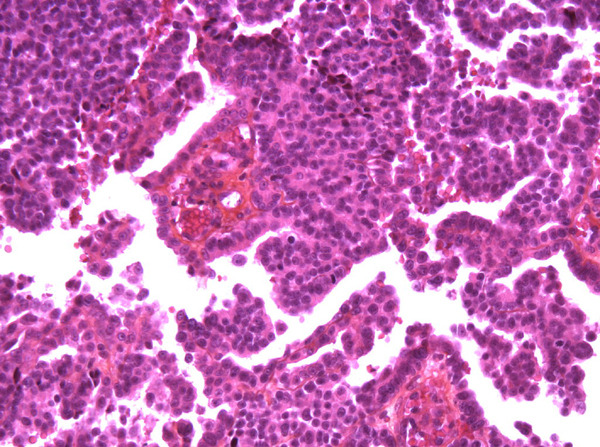
**Papillary pattern of mesothelioma, Hemalun Eosin Safran (HES)**.

**Figure 2:**
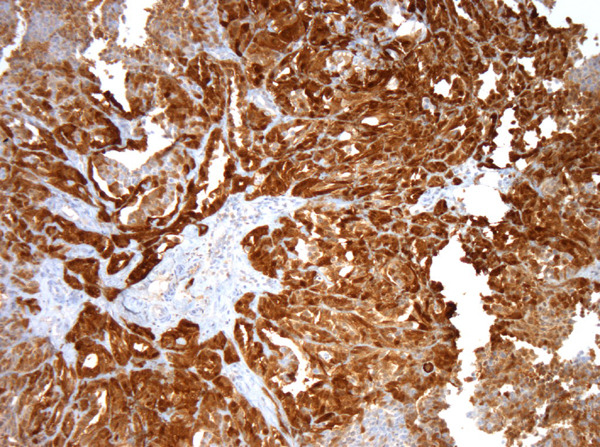
**Immunohistochemistry of mesothelioma, calretinin antibody, Zymec DC8, dilution 1/50**.

**Figure 3:**
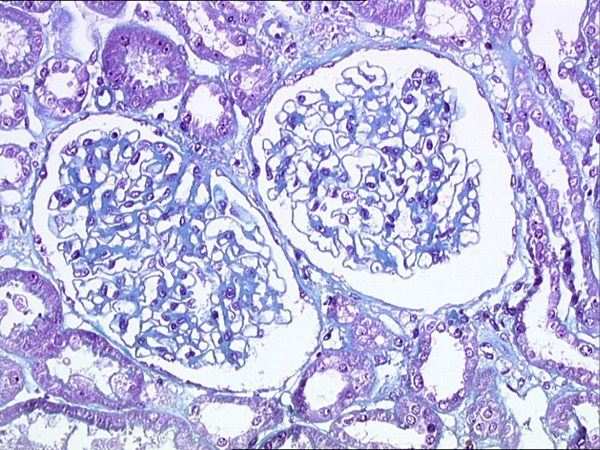
**Minimal change disease on the renal biopsy, Masson Trichrome (x200)**.

We report a 63-year-old Caucasian man diagnosed with a tumor of the right side of the scrotum in September 2002. He was operated through an inguinal incision. The aspect and histologic pattern were indicative of a tubulopapillar, malignant and well differentiated mesothelioma of the tunica vaginalis testis (Figures 1 and 2). Resection margins were invaded, thus a second surgical procedure was performed, with both a scrotectomy and an orchiectomy. A staging work-up, with thoraco-abdominal CT scan and standard blood chemistry showed no abnormality. The patient was then referred to our occupational medicine clinic. No exposure to asbestos was found, but he had a prolonged history of tobacco exposure for 30 years, until 1994. In August 2003, he experienced weight gain and generalized edema which regressed with furosemide and spironolactone diuretics. He was then referred to our institution for evolution of retroperitoneal lymph nodes on whole-body CT scan. The clinical examination was uninformative and there was no hypertension. The only abnormalities on the CT scan were 18 mm transversal inter aortico-cava and retro-cava lymph-nodes. Major biological abnormalities were seen in routine laboratory tests: low total serum protein (48 g/l), low serum albumin (9·7 g/l) and elevated cholesterol (5·59 g/l). However, serum creatinine was normal at 70 umol/l; liver enzymes, serum ionogram and triglycerides were within normal limits. Proteinuria was 9 g/24 hours without microscopic hematuria. The diagnosis of pure nephrotic syndrome led to performing a percutaneous renal biopsy in October 2003. It showed minimal change disease with neither immunoglobulins nor complement deposits (Figure 3). Other etiologies of nephrotic syndrome were eliminated: antibodies against hepatitis B and C viruses and against HIV, antinuclear antibodies and antineutrophil cytoplasmic antibodies were negative. Serum levels of C3, C4 and CH50 were normal. Oral prednisolone administration was initiated at a dose of 1 mg/kg/day. Furosemide was continued at a dose of 40 mg/day. His proteinuria disappeared in December 2003, so the prednisolone dosage was progressively decreased. While the nephrotic syndrome became controlled, retroperitoneal disease progressed both in size and localisation. A control CT scan performed in February 2004 showed increased lombo-aortic lymph nodes, with a suspicion of extension to the retrocrural area. A percutaneous retroperitoneal lymph node biopsy showed tissue invasion by mesothelioma. As the nephrotic syndrome was well controlled by treatment, the strategy was to perform radical bilateral retroperitoneal lymph node dissection. There was no peritoneal involvement. The histologic aspect was epithelioid mesothelioma with necrosis and invasion of both the capsule and small vessels. A recurrence of the nephrotic syndrome was observed 10 days after the surgery. Prednisolone was then increased to 1 mg/kg/day for 1 month, and then decreased to 0.75 mg/kg/day, and then 0.5 mg/kg/day to allow for postoperative healing. In May 2004, a CT scan showed a disease progression with suprarenal lymph nodes of 20 mm maximal diameter. A combination of cisplatin 75 mg/m^2^ and pemetrexed 500 mg/m^2^ was initiated. Six cycles were given from June 2004 to September 2004. In June 2004, the renal function was normal, with a normal proteinuria, total serum protein of 59 g/l and serum creatinine of 75 umol/l. A slow decrease in prednisolone dosage over 6 months was decided. In September 2004, an elevated blood pressure was observed for the first time (systolic at 150 mmHg and diastolic at 95 mmHg), and serum creatinine levels increased to 125 umol/l. This was attributed to cisplatin renal toxicity, and then prednisolone decrease was continued. In October 2004, the CT scan showed disease progression in the retroperitoneum; FDG-PET examination revealed a unique site of radio-isotope fixation on the eleventh dorsal vertebra. A percutaneous biopsy showed involvement of mesothelioma, but the histologic pattern of the lesion was undifferentiated. Radiotherapy on the lumbar area was then decided. In November 2004, the renal function was normal and the nephrotic syndrome did not recur while he was receiving a daily dose of 0.1 mg/kg prednisolone. In February 2005, he developed disease recurrence with ascites. Cytological examination of the ascites showed mesothelioma involvement. Performance status declined, palliative treatment was given and the patient eventually died of disease progression in March 2005.

## Discussion

Paraneoplastic glomerulopathy is a well known entity [1] rarely associated with mesothelioma. A PubMed search using the keywords "mesothelioma", "paraneoplastic glomerulopathy" and every histopathologic subtype of glomerulopathy was performed. Only five cases of concurrent mesothelioma and nephrotic syndrome have been reported in the literature. All were associated with pleural mesothelioma [2-6] and all five patients were men. A diagnosis of nephrotic syndrome preceded mesothelioma in two cases and was concomittant in the three other cases. Membranous nephropathy, minimal change disease and membrano-proliferative glomerulonephritis were observed. Three patients had asbestosis exposure and only one patient with nephrotic syndrome was treated successfully with corticosteroids. All the five patients died of disease progression. As far as we know, this is the first report of a mesothelioma of the tunica vaginalis testis associated with nephrotic syndrome [7]. Nephrotic syndrome is often associated with membranous glomerulopathy in patients with solid tumors, especially in patients with lung and gastrointestinal tract neoplasias[8]. IgA nephropathy, minimal change disease and glomerulosclerosis are less frequent. The nephrotic syndrome usually precedes the tumor by several months according to Lee et al. [9] and renal disease antedates the diagnosis of cancer in two-thirds of the surveyed population. The diagnosis of paraneoplastic nephrotic syndrome may be evoked when the following criteria are present [10]: no evidence of other etiology of the nephrotic syndrome; time relationship between the diagnosis of nephrotic syndrome and cancer; tumor treatment associated with a decrease of renal symptoms; tumor recurrence associated with an increase in renal symptoms and proteinuria. A causal relationship is suggested if nephrotic proteinuria develops either 6 months before or after the diagnosis of malignancy. In the present case, we can discuss the paraneoplastic etiology of the nephrotic syndrome on four unusual elements: we found minimal change lesions, not a membranous nephropathy; the glomerulopathy was very sensitive to corticosteroids; the nephrotic syndrome occurred eleven months after the diagnosis of the primary malignancy, but concomitantly with the recurrence and; the nephrotic syndrome did not decrease with tumor control and did not recur when the mesothelioma escaped treatment. No other etiologies could explain this phenomenon.

## Conclusion

We conclude that this minimal change nephropathy is a casual event in the history of a very rare tumor. A person known to suffer from malignancy and who develops a nephrotic syndrome, should undergo renal biopsy if their general condition allows. The management of people with cancer and paraneoplastic nephropathy should focus on the following elements [8]. First, symptomatic treatment of the nephrotic syndrome with sodium and water restriction and diuretic therapy is justified. In the majority of patients, the use of a distal diuretic is sufficient. To our knowledge, there are no studies of corticotherapy in paraneoplastic glomerulopathies in the literature. Prophylaxis and the early treatment of complications of the nephrotic syndrome such as thromboembolism, infections and lipid abnormalities are useful. ACE inhibitors can be used to decrease blood pressure and proteinuria, controlling hyperkalemia and renal function. Second, a systematic search for associated electrolyte abnormalities is legitimate. Third, all treatments should be regularly analyzed to avoid further toxicity; drugs binding albumin may be used with caution. And last, the treatment of neoplasia should be undertaken rapidly. Patients with cancer may be screened daily for proteinuria at diagnosis and during the course of the disease.

## Abbreviations

HES: Hemalun Eosin Safran.

## Competing interests

The authors declare that they have no competing interests.

## Consent

As the patient died three years ago, we could not obtain his consent for publication of results. We tried but were unable to trace his next of kin. However, the patient cannot be identified and we see no reason why his next of kin would object to publication of this case report.

## Authors' contributions

JB wrote the manuscript and reviewed the literature about paraneoplastic glomerulopathies. DR and FD perfomed pathology examinations and provided figures (mesothelioma and kidney biospy, respectively). JPD perfomed the revisions and the final approval of the manuscript.
